# The interplay between sleep and ecophysiology, behaviour and responses to environmental change in fish

**DOI:** 10.1242/jeb.247138

**Published:** 2024-06-11

**Authors:** Helena Norman, Amelia Munson, Daphne Cortese, Barbara Koeck, Shaun S. Killen

**Affiliations:** School of Biodiversity, One Health, and Comparative Medicine, University of Glasgow, Glasgow G12 8QQ, UK

**Keywords:** Circadian rhythms, Climate change, Metabolism

## Abstract

Evidence of behavioural sleep has been observed in every animal species studied to date, but current knowledge of the behaviour, neurophysiology and ecophysiology associated with sleep is concentrated on mammals and birds. Fish are a hugely diverse group that can offer novel insights into a variety of sleep-related behaviours across environments, but the ecophysiological relevance of sleep in fish has been largely overlooked. Here, we systematically reviewed the literature to assess the current breadth of knowledge on fish sleep, and surveyed the diverse physiological effects and behaviours associated with sleep. We also discuss possible ways in which unstudied external factors may alter sleep behaviours. For example, predation risk may alter sleep patterns, as has been shown in mammalian, avian and reptilian species. Other environmental factors – such as water temperature and oxygen availability – have the potential to alter sleep patterns in fish differently than for terrestrial endotherms. Understanding the ecological influences on sleep in fish is vital, as sleep deprivation has the potential to affect waking behaviour and fitness owing to cognitive and physiological impairments, possibly affecting ecological phenomena and sensitivity to environmental stressors in ways that have not been considered.

## Teaser

**Summary:** This Review summarises the current knowledge on fish sleep behaviour in an ecophysiological context, and suggests future directions for this field in a changing world.

## Introduction

Sleep is a neurophysiological state that is widespread throughout the animal kingdom (Rattenborg and Ungurean, 2023). From an ecological perspective, sleep is a state of disengagement from the environment, whereby an individual foregoes foraging, mating or being vigilant, and instead enters a potentially dangerous state of deceased sensitivity to external stimuli, increasing vulnerability to potential external threats (Allen, 2011; Tworkowski and Lesku, 2019). From a neurological perspective, it is a complex and tightly regulated process that shows remarkable evolutionary conservation (Allada and Siegel, 2008). Despite the enormous evolutionary distances and physiological differences among organisms studied, commonalities in sleep behaviour have been found across taxa, from invertebrates – such as jellyfish (Lesku and Ly, 2017; Nath et al., 2017), hydra (Kanaya et al., 2020), flatworms (Omond et al., 2017) and *Drosophila* (Hendricks et al., 2000; Shaw et al., 2000) – to vertebrates, including fish (Lesku et al., 2019), amphibians (Libourel and Herrel, 2016), reptiles (Shein-Idelson et al., 2016), birds and mammals (Lesku et al., 2019). The near-ubiquitous occurrence of sleep behaviours, even in species without brains (e.g. jellyfish; Rattenborg and Ungurean, 2023), suggests an ancient, common neural signature. Indeed, many of the cellular and genetic processes controlling sleep have been conserved throughout evolutionary history (Allada and Siegel, 2008; Aulsebrook et al., 2016; Bringmann, 2018; Jaggard et al., 2021; Lesku et al., 2019). Although studying sleep across taxa is useful for comparing its physiology and behaviours, some taxa are frequently overlooked, such as fishes (Rattenborg et al., 2022). The diversity and abundance of fish species that inhabit a variety of different habitats could provide new understanding of the function, evolution, mechanisms and plasticity of sleep (Keene and Appelbaum, 2019).

**Glossary**

**Aerobic metabolic scope**

The difference or ratio between the maximum metabolic rate and standard metabolic rate; represents the capacity of an animal to supply oxygen to tissues for aerobic metabolic processes above that required for maintenance including growth, activity and digestion.

**Arousal threshold**

The minimum intensity of stimulus necessary to elicit a response.

**Circadian system**

The molecular and behavioural changes in an organism over a 24-h cycle.

**Motor automatisms**

Small movements carried out by an unconscious individual.

**Rapid eye movement (REM) sleep**

A phase of sleep characterised by fast eye movements in mammals and birds, accompanied by low muscle tone.

**Sleep architecture**

The combination of sleep duration, frequency of disruptions, length of disruptions and – in species exhibiting REM/non-REM sleep – the relative time spent in each sleep type.

**Superficial sleep**

The transition period between wakefulness and sleep, in which the arousal threshold is substantially lowered.

**Two-process model of sleep regulation**

A conceptual framework that posits sleep is controlled by both homeostasis and the circadian clock, and interactions between the two.

**Unihemispheric sleep**

Sleep occurring in one hemisphere of the brain while the other hemisphere is awake.

Early research on sleep excluded fish, and the literature at the time referred to sleep states in fish as ‘rest’ or ‘sleep-like states’ (Meddis, 1975). ‘Sleep’ was defined by the presence of slow-wave cortical activity (Cabanac et al., 2009); however, this definition excluded (among other animals) fish, which lack mammalian cortical structures (Allen, 2011). Similarly, rapid eye movement (REM; see Glossary) sleep excludes animals that have evolved eye movement control that is different from that of mammals (Árnason et al., 2015). By excluding species owing to non-transferable physiological criteria, it becomes impossible to perform phylogenetic comparisons to better understand sleep. To expand the definition of sleep to be inclusive of non-mammalian and non-avian species, Campbell and Tobler (1984) created a set of criteria to describe behavioural sleep, which did not rely on taxa-specific physiological parameters. These criteria are: (1) a state of behavioural quiescence regulated by the circadian system (see Glossary); (2) an increased arousal threshold (see Glossary) and reduced responsiveness to external stimuli; (3) a species-specific sleeping site and/or posture; and (4) if criteria 1–3 are fulfilled, they must exhibit rapid state reversibility, so as to exclude comas, torpor and anaesthesia. This pioneering review opened a new field in the evolution, ecology and function of sleep across taxa, specifically in species that had been previously neglected. The group of defining criteria has since expanded to include homeostatic regulation, such that sleep deprivation results in a subsequent period of compensatory sleep (Aho et al., 2017; Tobler, 2011; Tobler and Borbely, 1986; Yokogawa et al., 2007; Zada and Appelbaum, 2020; Zhdanova, 2011; Zhdanova et al., 2001).

The fact that fish have historically been overlooked in the field of sleep research can, in part, be attributed to the difficulties in distinguishing true sleep from rest behaviour in these species. The difficulty in applying technologies that have been developed for use in terrestrial animals to aquatic taxa has only exacerbated these challenges (Zada and Appelbaum, 2020). Throughout the literature, ‘sleep’ has often been used as a default term to refer to resting behaviour, without ensuring that all criteria of behavioural sleep have been met; this is especially common in the study of non-model organisms. Despite the complications in studying fish sleep, this vital behaviour should not go unresearched, as the potential insights it offers into fish waking behaviour and physiology may be invaluable for furthering our understanding of behavioural ecology, ecophysiology and responses to environmental change.

In this systematic Review, we synthesize the criteria of sleep examined in different fish species. We begin by outlining the methods by which we selected papers to include in this Review, and go on to discuss what these papers reveal about each of the four criteria listed above. We discuss the novel behaviours that accompany such criteria, and the findings and implications of studying sleep in fish models for fields ranging from evolutionary biology to pharmacology. We also consider evidence for the ecological relevance of sleep in fish, including how various biotic and abiotic factors may affect sleep, and how sleep – or a lack thereof – may affect fish behavioural ecology and ecophysiology. Our aim is to summarise the evidence for sleep in fish, as well as to identify current knowledge gaps and priorities for future research.

## Survey of the literature

**Fig. 1.**
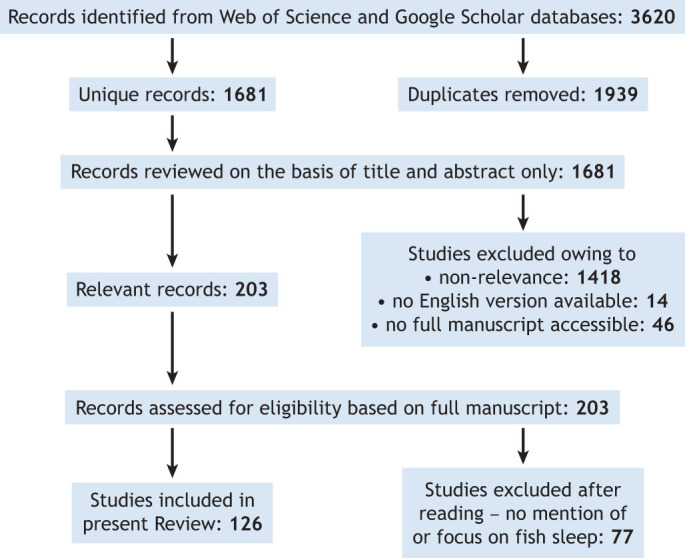
**Schematic outlining the screening process, whereby papers were eliminated or chosen for inclusion in the systematic review based on eligibility.** Relevant papers included at least one behaviour needed for sleep or recorded the tested absence of any behaviours. One study was found in references that had not been found by this search process, and it was included after the eligibility screen.

To select studies to include in this Review, we conducted a systematic literature search using the global search engines Web of Science and Google Scholar, using the search terms outlined in Table 1. We then systematically sorted through the records using the methodology outlined in Fig. 1. From an initial 3620 papers obtained from the searches, and after a detailed screening of 203 full papers for eligibility, we found a total of 126 relevant papers. Of these, 82 were primary research papers (both peer-reviewed and doctoral theses) and 44 were review articles, focusing either on fish (mostly from a neuroscience perspective) or on sleep across taxa with a mention of fish (see Table S1).

**Table 1.**
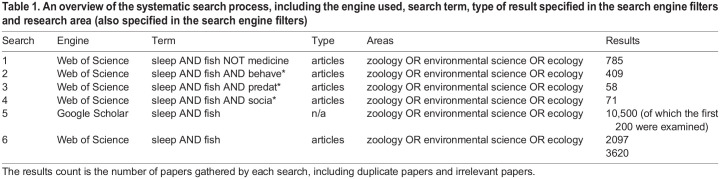
An overview of the systematic search process, including the engine used, search term, type of result specified in the search engine filters and research area (also specified in the search engine filters)

**Fig. 2.**
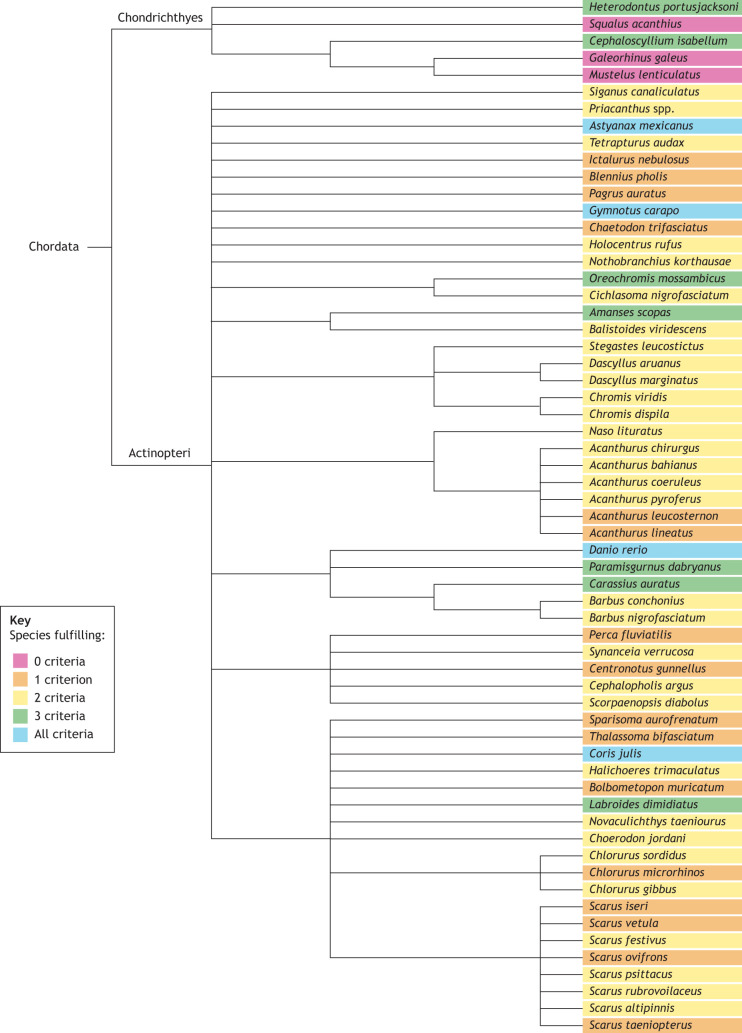
**An unscaled phylogenetic tree showing all fish species exhibiting any criteria for behavioural sleep, or found to exhibit none.** Species are colour-coded to show the number of criteria observed or experimentally found in each species (0 to 4). Species fulfilling less than four criteria are not incapable of fulfilling all criteria for sleep but may not have been empirically studied for all criteria; thus, this phylogeny serves to highlight the disparity between the number of species described to ‘sleep’ in some capacity and the actual study of sleep behaviour. Species fulfilling zero criteria have been shown to not fulfil any criteria of sleep, which is discussed further in the section ‘Evolution of sleep’. Criteria are: (1) circadian-regulated periods of quiescence, (2) an increased arousal threshold, (3) species-specific posture and (4) homeostatic regulation.

**Fig. 3.**
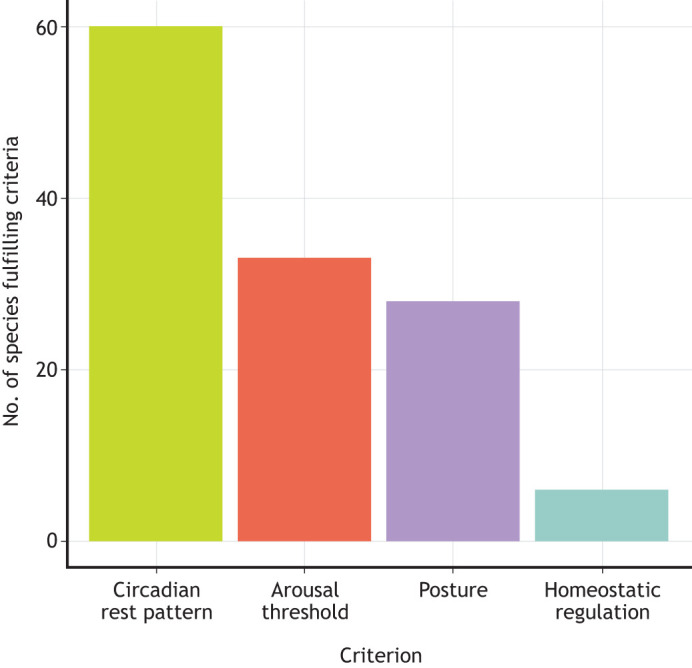
**Bar graph showing the number of fish species that have been recorded as fulfilling each of the four criteria of sleep across the literature examined in this Review.** Some species have been observed performing more than one sleep-associated behaviour, and such species are represented separately within each relevant category in the figure.

Sleep-like states in fish have been studied since 1955 (Qasim, 1955); however, studies focusing on sleep in fish have rapidly increased in number in the past two decades, with the majority focusing on neurophysiology associated with sleep. Zebrafish (*Danio rerio*) are the most extensively studied fish species with regards to sleep (Norton and Bally-Cuif, 2010; Oikonomou and Prober, 2017), owing to their suitability as a laboratory species, and their parallels with aspects of human biology (Volkoff, 2012; Zhdanova, 2006, 2011). Although many studies discussed in this Review focus on zebrafish, some examine behaviours associated with sleep in a wider range of species, but typically consider only one or two of the sleep criteria, such as daily patterns of behaviour. Overall, only a few fish species have been empirically tested against all criteria for sleep: *Danio rerio*, *Astyanax mexicanus*, *Gymnotus carapo* and *Coris julis*. A further seven species have been tested against all behaviour criteria, excluding sleep homeostasis: *Heterodontus portusjacksoni*, *Cephaloscyllium isabellum*, *Paramisgurnus dabryanus*, *Carassius auratus*, *Amanses scopas*, *Labroides dimidiatus* and *Oreochromis mossambicus*. Although many studies identified in this Review use the term ‘sleep’ to describe a resting behaviour, most did not test fish against all criteria for behavioural sleep, and defaulted to describing sleep based on as little as one behavioural criterion (Fig. 2), owing to methodological limitations. Of course, studies that only record partial aspects of sleep behaviour are still hugely important, as they form a foundation of knowledge on the diversity of sleep-associated behaviours. The sleep homeostasis criterion is particularly challenging to assess, because it has so far only been measured through experimental means, limiting the work on species typically studied in the wild. This is reflected in how few species have fulfilled this criterion (Fig. 3). Additionally, some studies report a sleep-associated behaviour incidental to the study; for example, circadian activity/rest is the most-researched criterion of sleep in fish, but often these studies only look at circadian movements, and the circadian-regulated rest aspect is studied only for understanding the timing of other behaviours. The fragmented nature of knowledge on fish sleep highlights the extent to which sleep has been overlooked in fish research.

## Observational and experimental evidence of the four sleep criteria in fish

### Circadian-regulated behavioural quiescence

The first criterion of behavioural sleep is undoubtedly the most extensively studied, as the predictable 24-h cycle of light and dark affects most organisms, and is meaningful for behaviours outside of sleep (Allen, 2011). Many animals have evolved cycles of activity and quiescence in accordance with the daily photoperiod, which is a major ‘zeitgeber’ (German for ‘timekeeper’) (Reebs, 2002; Steindal and Whitmore, 2019). The observed variation in activity and rest is tied to the production of melatonin, a molecule that is hugely important for the synchronisation of sleep–wake cycles (Gandhi et al., 2015; Perry, 2022). Genes coding for melatonin production respond to the outputs of pineal photodetection cells (Kuz'mina, 2020), with changes in photoperiod affecting melatonin synthesis (Perry, 2022), and the effects of melatonin production differing between diurnal and nocturnal species (López-Olmeda et al., 2006). It should be noted, however, that this first criterion, and the regulation of sleep as whole, is intrinsically tied to sleep homeostasis. In mammals, the two-process model of sleep regulation (see Glossary) ties together the intrinsic circadian molecular clock and sleep debt (Borbély, 1982; Borbély et al., 2016).

**Fig. 4.**
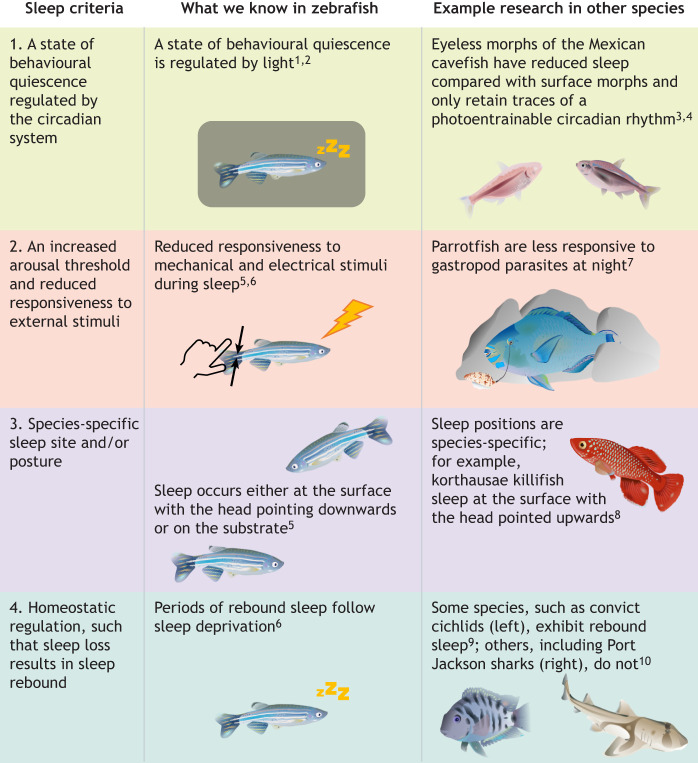
**Schematic showing behavioural criteria for sleep, with insights from fish research.** The sleep criteria were defined by Campbell and Tobler (1984) and built on by Tobler and Borbely (1986). Insights are shown from research on the model organism *Danio rerio*, and other fish species studied in the context of each criterion. References: 1: Sigurgeirsson et al. (2013); 2: Pinheiro-da-Silva et al. (2018); 3: McGaugh et al. (2020); 4: Duboué et al. (2011); 5: Zhdanova et al. (2001); 6: Yokogawa et al. (2007); 7: Johnson et al. (1995); 8: Lucas-Sánchez et al. (2013); 9: Tobler and Borbely (1986); 10: Kelly et al. (2020a).

Zebrafish have specifically been shown to fulfil the first criteria for sleep, as a diurnal species that naturally sleeps during the night. Altering the primary zeitgeber (i.e. light) in laboratory experiments disrupts the circadian rhythm, thus depriving zebrafish of sleep (Pinheiro-da-Silva et al., 2017a,b, 2018; Sigurgeirsson et al., 2013) (Fig. 4, row 1), although this method of sleep deprivation is highly stressful and disrupts the entire circadian system. Other zeitgebers that synchronise biological rhythms include temperature, predation risk and food availability (Myers et al., 2016; Reebs, 2002).

Although photoperiod is key for the entrainment of the endogenous circadian system in many fish, some species maintain circadian rest–activity patterns without a photoperiod. Fish living in constant dark conditions in the wild, such as the Mexican cavefish (*Astyanax mexicanus*), have provided unique insight into endogenous circadian clocks when studied under light–dark conditions. There are two distinct morphs of Mexican cavefish: one that lives at the surface and possesses eyes, and another that lives deeper in caves and is eyeless (Duboué et al., 2011). The blind morph evolved independently 29 times in different cave populations (Jaggard et al., 2017; Mack et al., 2021), and where hybridisation occurs, there is constant selection against surface morph traits (Borowsky, 2023). Alongside the phenotypic changes between morphs, such as blindness and scale pigmentation, there are also functional differences relating to their sleep: eyeless forms have evolved to sleep very little – typically only 2 h per 24-h period (Duboué et al., 2011; McGaugh et al., 2020; Yoshizawa et al., 2015) – whereas the surface morphs sleep around 8 h per 24-h period (Duboué et al., 2011; Yoshizawa et al., 2015), with hybrids exhibiting an intermediate amount of sleep (Moran et al., 2022). The reduction in sleep in blind fish has been hypothesised to be due to the sensory processing changes that accompany life in complete darkness (Jaggard et al., 2017), and are likely to be driven by an upregulation in hypocretin signalling (Jaggard et al., 2018). Importantly, however, surface morphs have endogenous circadian clocks, which regulate metabolism with photoperiod, increasing the oxygen demand during the daytime. This endogenous circadian rhythm prevails under constant dark conditions as well: surface fish exhibit the same fluctuations in metabolism without photoperiodic cues under experimental conditions (Moran et al., 2014). In the same constant dark conditions, however, blind morphs do not have a daily cycle in their metabolic rate. This is likely due to the fact that these fish experience a lesser degree of environmental variation over the course of a day, which is likely to result in energy savings compared with surface morphs (Fig. 4, row 1) (Moran et al., 2014), which is beneficial in nutrient-poor cave environments. Other, less extensively studied fish species, such as the Somalian cavefish (*Phreatichthys andruzzii*) and the several hillstream loach species of the family Balitoridae, have adapted similarly to life in caves*.* These species have convergently evolved populations with no eyes and a greatly reduced need for sleep compared with their surface-living counterparts (Cavallari et al., 2011; Duboué and Borowsky, 2012). Interestingly, weak remnants of zeitgeber-entrainable chronobiological rhythms are consistent across convergently evolved species, whereby cave morphs exhibit behavioural fluctuations in response to regular photoperiod or food provisioning (Carlson and Gross, 2018; Cavallari et al., 2011; Duboué and Borowsky, 2012).

Observational studies in the field and lab have recorded 24-h patterns of activity and quiescence in a variety of fish species. Although the other criteria for sleep have often not been tested in these species, an understanding of these patterns is still useful generally to understand sleep across taxa. Catfish (*Ictalurus nebulosus*) undergo a nocturnal period of rest, during which they exhibit periods of sleep with motor automatisms (see Glossary) and a reduction in heart rate (Belich, 1984). Similarly, neon and cardinal tetras (*Paracheirodon innesi* and *P. axelrodi*) become quiescent at night (Lythgoe and Shand, 1983). *Paramisgurnus dabryanus*, a loach species, decrease activity and ‘float on the water’ more at night (Zhang et al., 2022); this ‘floating’ behaviour is accompanied by decreased sensitivity to external stimuli and decreased respiratory frequency. Of course, not all fish are diurnal. For example, nocturnal patterns of activity and rest have been observed in draughtsboard sharks (*Cephaloscyllium isabellum*), which become quiescent in the day, exhibiting a lower metabolic rate and a flat body posture (Kelly et al., 2022). Circadian-mediated rest has also been observed in two species of buccal-pumping sharks (*Heterodontus portusjacksoni* and *Cephaloscyllium isabellum*), and when the photoperiod is altered, the activity rhythms of both species are disrupted (Kelly et al., 2020a). All circadian rhythms are subject to selection, although the timing and duration of sleep can change plastically in response to environmental cues. Additionally, selection on sleep patterns is often accompanied by selection on physical traits; nocturnal cichlids, for example, evolve larger eyes for enhanced nighttime visual processing (Lloyd et al., 2021). Studying closely related species, or subpopulations of the same species, with differing diel activity patterns may be important to further our understanding of the mechanisms behind nocturnality.

Understanding chronobiology in fish allows for comparison of general patterns across taxa. For example, in fish, as is seen in other animal taxa (Chow et al., 2016; Garau et al., 2006; Güldür and Otlu, 2017; Mortola, 2007), the circadian clock also regulates the daily fluctuations of energy metabolism (Guinea and Ferniandez, 1991; Kelly et al., 2021) and respiration rate (Thetmeyer, 1997). This alludes to one function of sleep: it can be considered as a means of slowing some cellular processes to conserve energy. Similarly, across taxa, the endogenous circadian clock becomes impaired with age (Xu and Li, 2022; Zhao et al., 2019). This is observed in *Nothobranchius korthausae*, whereby senescent fish do not maintain their endogenous rhythm in the absence of a light zeitgeber (Lucas-Sánchez et al., 2013). Additionally, in aged zebrafish, the usual rhythms of rest and activity become desynchronised with the 24-h clock in the absence of light and dark cues (Zhdanova et al., 2008).

### Increased arousal threshold

The second criterion for sleep, an increased arousal threshold, has been demonstrated for several fish species. Zebrafish exhibit reduced sensory responsiveness to both mechanical stimuli (measured as the number of stimuli required to initiate locomotion; Zhdanova et al., 2001) and electrical stimuli (measured as the voltage required to induce a response; Yokogawa et al., 2007) when engaged in a species-typical sleeping posture (Fig. 4, row 2). Mozambique tilapia (*O. mossambicus*) also display lessened sensitivity to both electrical and food stimuli when they appear to be asleep (Shapiro and Hepburn, 1976). Divers investigating nocturnal activities in large aquariums, pre-dating the formal definition of sleep, identified this environmental disengagement and realized that they could lift individual fish from the bottom of the tank when they appeared to be asleep (Russell, 1971).

Species that interact with fish can take advantage of these periods of environmental disengagement. For example, cleaner shrimp (*Urocaridella antonbruunii*) eat dead scales and debris from rabbitfish (*Siganus canaliculatus*) during the night (Bos and Fransen, 2018); although this is a mutualistic relationship, rabbitfish can still occasionally eat cleaner shrimp, so by engaging in this behaviour when the fish are sleeping, cleaner shrimp reduce their likelihood of being eaten. Gastropod molluscs also exploit the higher arousal threshold in sleeping Scaridae fishes and parasitise them as they sleep, inserting their proboscises through their eyes or mouth (Fig. 4, row 2; Bouchet, 1989; Johnson et al., 1995). Humans take advantage of the increased arousal threshold in fish for surveying purposes: researchers have dropped video cameras underwater while fish are in this state of decreased arousal to obtain population estimates of *Pagrus auratus* (Morrison and Carbines, 2006). The reduced sensory responsiveness associated with sleep means that fish species often seek protection during the night in the form of sleeping sites. From the perspective of predation risk, decreased sensory awareness and responsiveness could put individuals at a greater risk of being captured by a predator, given that their escape abilities are compromised while in the sleep state. This would suggest that the evolutionary benefits of sleep must be significant to overcome the potential increase in predation risk, or that some degree of sleep is physiologically unavoidable. Alternatively, it could be argued that increased sleep could vastly decrease predation risk: sleep might allow individuals to reduce their overall activity level or even hide while incurring a minimal energetic cost, thus reducing their likelihood of encountering a predator or drawing attention to themselves (Lima and Rattenborg, 2007). This parallels the well-known trade-off between foraging activity and predation risk. In other taxa, the risk of predation increases vigilance and decreases sleeping behaviour (Dominguez, 2003; Lendrem, 1984; Revell and Hayes, 2009), and a similar trade-off could be true in fish (Fox and Donelson, 2014). Overall, the exact importance of sleep for predation risk is likely to be species-dependent in fishes, and is an area that requires additional study.

### Species-specific posture and/or site

The third criterion used to identify behavioural sleep is that species must adopt a specific posture and/or site for undertaking sleep. For example, some marine fish make use of corals in this capacity. Some fish species are specific to certain coral types; for example, *Abudefduf saxatilis*, *Acanthurus coeruleus* and *Microspatodon chrysurus* take refuge in fire-branching corals during the night (Coni et al., 2012). *Amanses scopas*, the filefish, also shows a preference for fire-branching corals; however, instead of seeking refuge, they anchor to the coral with their teeth (Eyal et al., 2011). Using the structural safety provided by the coral to reduce night-time predation or parasitism does not come without risk: fish taking refuge in corals may be exposed to hypoxia when corals respire at night. To mitigate the effects of low oxygen concentrations, some species have developed unusual sleep behaviours, fanning their fins to increase aeration of the corals while they sleep (Goldshmid et al., 2004). Other fish species opt for using holes and nooks in the reef as sleeping sites, whereas some build their own shelter. For example, *Novaculichthys taeniourus* and *Choerodon jordani* both construct nest piles from coral fragments and rubble (Nanami and Nishihira, 1999; Takayanagi et al., 2003). There are also species that bury themselves in sand, such as *Halichoeres trimaculatus* (Hur et al., 2012) and *Coris julis* (Videler, 1988). Field observations have been supported by behaviour seen in captive fish: *Labroides dimidiatus* in aquaria exhibits similar behaviour to fish in the wild, using a sleeping cave that mimics natural reef structure (Lenke, 1988). As sleep sites are valuable commodities for many species, competition often occurs for the best sites (Shulman, 1985), and individuals defend their sites from others (Robertson et al., 1979). For example, monogamous butterflyfish (*Chaetodon trifasciatus*) pairs defend a sleeping territory, which periodically becomes their spawning site (Yabuta, 1997). Some species do not compete over sites, but migrate daily between their foraging site and a secondary sleeping site, such as a nearby shallow coral (Gomi et al., 2021), reef slope (Welsh and Bellwood, 2012) or deeper waters (Robertson et al., 1979). These migrations are all aligned with photoperiod: fish leave their diurnal range before dark and remain in their sleeping location all night (Welsh and Bellwood, 2012).

Species-specific sleep postures have been observed and described in a small number of fish species. For example, zebrafish sleep either while floating, with their heads pointed downwards, or at the bottom of the tank, in a horizontal posture (Fig. 4, row 3) (Zhdanova et al., 2001). Cardinal tetras (*Paracheirodon axelrodi*) have anecdotally been observed to sleep at the bottom of the tank (Lythgoe and Shand, 1983), and banded knifefish (*Gymnotus carapo*) sleep with their heads up, at an angle of 30 deg relative to the bottom of the aquarium (Stopa and Hoshino, 1999). Additionally, several *Barbus* species tip at a 45–60 deg angle (Barlow, 1976), and *Nothobranchius korthausae* sleeps at the top of an aquarium with its head tipped upwards (Fig. 4, row 3) (Lucas-Sánchez et al., 2013). Whether these specific sleep postures are accompanied by the use of specific sleep sites is unknown, as their observation is mostly limited to laboratory settings. However, distinct sleeping postures have been documented in aquafarms, such as the floating behaviour of *Paramisgurnus dabryanus* (Zhang et al., 2022), and in wild populations, such as the shallow-water aggregations observed in sleeping bumphead parrotfish (*Bolbometopon muricatum*) (Muñoz et al., 2014).

### Homeostatic regulation

The fourth and final criterion of sleep is that the state must be homeostatically regulated, so if sleep is reduced in quality or duration on any night, it is compensated for in the future. That sleep deprivation is followed by rebound sleep has been observed in several fish species including zebrafish, the convict cichlid, *Cichlasoma nigrofasciatum* (Fig. 4, row 4), and the goldfish, *Carassius auratus* (Tobler and Borbely, 1986). However, studies in zebrafish have shown that the presence of rebound sleep is dependent on the method of sleep deprivation (Fig. 4, row 4; Aho et al., 2017; Yokogawa et al., 2007). Although animals living in darkness may not regulate periods of quiescence through the circadian rhythm, there is evidence of homeostatic control of sleep remaining intact: both surface and eyeless forms of *Astyanax mexicanus* show rebound sleep after a period of shaking-induced sleep deprivation (McGaugh et al., 2020). However, two species of shark, *Heterodontus portusjacksoni* and *Cephaloscyllium isabellum*, do not show signs of rebound rest following a short period of rest deprivation, which sheds some doubt onto whether there is homeostatic control of sleep in these species (or, in fact, in sharks generally; Fig. 4, row 4; Kelly et al., 2020a). Alternatively, the period of sleep deprivation did not incur a great enough sleep debt for rebound sleep.

A plethora of correlative and some experimental studies indicate that the key functions of sleep are memory consolidation (Lee et al., 2020), cellular repair mechanisms (Zada et al., 2019), energy saving (Guinea and Ferniandez, 1991; Kelly et al., 2021) and the clearance of reactive oxygen species as by-products of metabolism (Malik et al., 2020). This suggests that lacking sleep results in the accumulation of physiological debt that, if not repaid/compensated for (by rebound sleep, for instance), may result in direct and indirect fitness effects. Zebrafish deprived of sleep by light exposure perform worse cognitively (Pinheiro-da-Silva et al., 2017a,b, 2018), supporting the hypothesised function of sleep to reduce neural load and replenish learning skills (Altenhofen and Bonan, 2022). Sleep-deprived zebrafish also show differences in their activity: they travel shorter distances and reach slower maximum speeds when partially or fully sleep deprived, compared with their counterparts with a full night of sleep (Pinheiro-da-Silva et al., 2018), and they show anxious behaviours (Singh et al., 2013). Inducing sleep deprivation through constant light exposure, however, is controversial and fraught with confounds, as it disrupts the circadian system entirely. Zebrafish sleep deprived by water flow at night, a less stressful and more ecologically relevant method of sleep deprivation, exhibit fewer startle responses relative to those exposed to constant water flow in the day. This indicates that sleep deprivation caused by less stressful methods still has next-day behavioural consequences in fish (Aho et al., 2017).

## Sleep and the environment

Studying how environmental features influence sleep in fish will help us to expand our knowledge of fish sleep specifically and will also broaden our understanding of the effects of ecological parameters on sleep. In other taxa, the social and physical environments have varying effects on sleep behaviour and architecture (see Glossary; Beauchamp, 2009; Christian et al., 1984; Dominguez, 2003; Kim and Monaghan, 2005; Revell and Hayes, 2009; Tisdale et al., 2018). Differences in aspects of sleep architecture (see Glossary) are well understood in certain fish study systems, such as in cave and surface morphs of cavefish species (Duboué et al., 2011); however, more nuanced differences between the sleep architectures of fish co-existing within a shared environment are less well known. Investigating the effects of environmental factors on sleep in fish could reveal important information about their physiology. For example, for ectotherms such as fish, temperature plays a key role in determining waking behaviours; studying the impact of elevated temperature on sleep in fish could reveal insights about energy allocation.

**Fig. 5.**
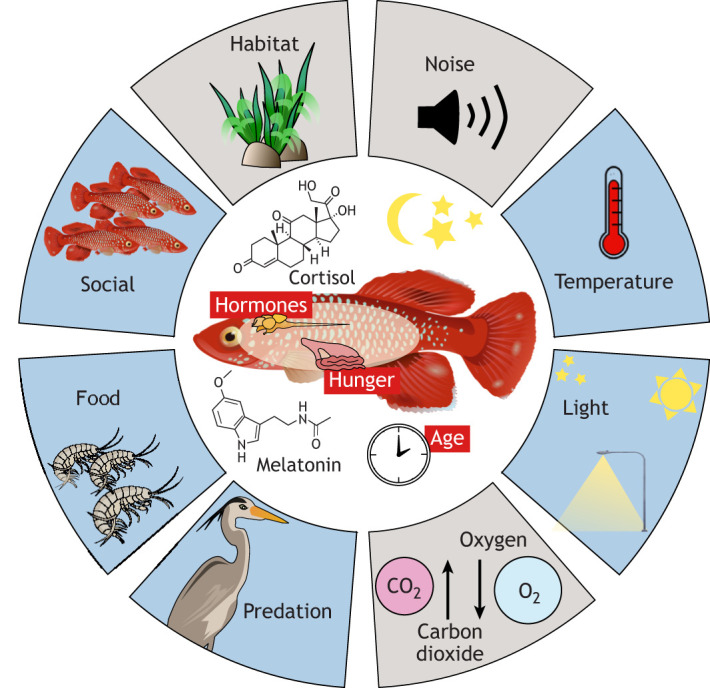
**Schematic depicting possible external and internal stimuli that influence individual sleep architecture and quality.** External stimuli are shown in the outer part of the circle, and internal stimuli are shown in red in the inner part. The colour code of external stimuli refers to whether there are studies looking at the effect of that factor on sleep in at least some fish species (blue) or whether studies are completely lacking (grey).

In aquaculture, environmental conditions such as photoperiod and temperature are manipulated to optimise fish growth and survival (Boujard et al., 1995; Li et al., 2021; Thyholdt, 2014). It would thus be relevant to know how varying these conditions affects fish sleep. Furthermore, a more nuanced understanding of sleep would be useful to inform laboratory holding conditions. Aside from the animal welfare considerations, understanding the potential for night-time lab conditions to disrupt sleep may be vital in determining the effects of sleep deprivation on behaviour or physiology in daytime experiments. As discussed above, sleep-deprived zebrafish have reduced cognition and display differences in locomotion (Pinheiro-da-Silva et al., 2017b, 2018), which could impact the results of studies investigating learning and movement. Moreover, constant sleep deprivation can increase mortality. In an experiment on *Lipophrys pholis*, larvae under constant light suffered higher mortality than those that experienced a normal photoperiod (Qasim, 1955). However, younger fish may be more susceptible to sleep loss-associated mortality as they sleep more than adults (Kayser and Biron, 2016). Similarly, in one study, two sand labris (*Coris julis*) exhibited erratic activity patterns and then died after being exposed to intensive constant light regimes (Videler, 1988). Typically, the primary concern when housing fish is the number of hours of light and dark exposure. In zebrafish, for example a 6-h night-time period is sufficient for homeostatic sleep, so lab conditions that ensure this amount of darkness should provide fish with sufficient time for adequate sleep (Zada et al., 2021); however, other factors affecting sleep are less understood, so although the temporal requirements for sleep may be satisfied, other factors may affect quality of rest. Thus, it is important to understand external factors that can cause changes in sleep patterns (Fig. 5), both in the lab and in the field, and then to determine (a) how changes in sleep patterns may induce a change in waking physiology and/or behaviour, and (b) what these changes may be.

Below, we consider the effects of variations in lighting, temperature, oxygenation, noise, food availability, shelter availability and predation risk on sleep in fish. Ultimately, the effects of different environmental effects on sleep in fish are likely to be complex and to interact with each other. To fully understand these effects, basic work understanding the effect of single and then multiple factors should be conducted across a range of fish species. Advancements in technology, such as infrared recording equipment and motion-detection software, have been crucial in allowing novel research into how fish sleep by enabling remote night-time recording. Further technological advancements will continue to open doors for research in this field. Rapidly advancing technologies for high spatial and temporal resolution of tracking fish in the wild (Guzzo et al., 2018; Matley et al., 2022) may also provide insight into the factors affecting circadian and sleep-related behaviours in fish and links with physiological traits at the individual level.

### Light

Exposure to light at night is well known to impair sleeping and circadian molecular function in diurnal species (Aulsebrook et al., 2018; Raap et al., 2016). Research on the effects of artificial light at night (ALAN) on fish suggests that increased light at night disrupts their circadian cycles such that many species of fish do not reduce their night-time activity under constant light conditions (Pulgar et al., 2019). This is in line with research on photo-entrainable circadian rhythms (Carlson and Gross, 2018; Duboué and Borowsky, 2012) and homeostatic regulation (Tobler and Borbely, 1986) in fish. Of course, ALAN is an increasingly relevant stressor in human-altered landscapes. The impact of ALAN on non-fish species – such as juvenile sea turtles trying to navigate to the ocean after hatching – has been highly publicised (Thums et al., 2016), and our understanding of the impacts on ecologically relevant behaviours in fish is growing. For example, nesting male smallmouth bass (*Micropterus dolomieu*) exposed to different patterns of light pollution exhibit altered nest defense behaviours (Foster et al., 2016). ALAN also increases the predation of larval marine fish in the daytime, potentially because of the effects of sleep deprivation, such as reduced swim speed (O'Connor et al., 2019; Pinheiro-da-Silva et al., 2018).

### Temperature

Many factors that have been shown to affect sleep in other taxa are still understudied in fish. Temperature is one such example, despite it being described as the ‘master factor’ (Brett, 1971) for determining ectothermic behaviour and physiology. Research in humans shows how temperature and sleep are intimately linked, with temperature extremes reducing sleep quality (Gilbert et al., 2004; Troynikov et al., 2018), but little is known about the direct effects of temperature on sleep architecture in fish. As ectotherms, fish are an intriguing system in which to study the link between temperature and sleep behaviour. Studies on other ectotherms, such as reptiles, found that sleep phase differs with brain temperature, showing a link between temperature and sleep architecture in terrestrial organisms (reviewed in Libourel and Barrillot, 2020).

Zebrafish and Nile tilapia (*Oreochromis niloticus*) both show a chronobiological rhythm in temperature preference, opting for warmer temperatures in the day, when these fish are more active (Vera et al., 2023). It is possible that temperature plays a role in the sleeping location selected by fish, with preference given to locations with a temperature that reduces sleep disruption or minimises potential trade-offs with energy allocation or risk of predation. Elevated temperature has been shown to increase activity in awake fish (Little et al., 2020), so if sleeping fish are exposed to higher temperatures, we might expect sleep disruptions, which could affect behaviour the following day (Pinheiro-da-Silva et al., 2017b, 2018) as well as alter aerobic metabolism and hormonal signalling (Christensen et al., 2021). Alternatively, if exposure to elevated temperature occurs during a non-sleep period and leads to fish becoming more active, we might expect to see increased sleep the following night to allow the fish to recover from periods of high activity, or the use of increased sleep as a means to reduce overall energy expenditure (Lesku and Schmidt, 2022). Increased sleep could have impacts on predation risk if fish are less responsive for longer periods; it may also reduce time for foraging the following day, which could compound the effects of warming, as temperature generally increases energy demand in fish (Neubauer and Andersen, 2019).

### Hypoxia

Although oxygen availability is rarely an issue in terrestrial ecosystems – with exceptions being at high altitudes (Anholm et al., 1992; Pappenheimer, 1977) – in aquatic ecosystems, hypoxic zones can be frequent and are increasing in intensity owing to human actions (Altieri et al., 2017; Diaz and Rosenberg, 2008; Jenny et al., 2016). Hypoxia is known to affect fish physiology and behaviour during waking hours, but the impact on sleep is less well understood, although the presence of fin-fanning behaviour in coral-dwelling species (discussed above) suggests that there is selective pressure to mitigate hypoxic conditions (Goldshmid et al., 2004). Under hypoxic conditions, fish aerobic metabolic scope (see Glossary) is decreased, which limits their aerobic swimming performance (Domenici et al., 2013; Fry, 1971). Therefore, fish may be less active and thus require less true sleep, but rest more; additionally, the decreased oxygen available per se may affect the quality of sleep (e.g. see studies on hypobaric hypoxia: Hale et al., 1984; Pappenheimer, 1977; Ray et al., 2011), with effects on their behaviour the next day. In mammalian systems, systemic hypoxia can in some cases be induced by sleep apnea, which results in a decrease in blood oxygen levels during sleep. This causes disruption of normal sleep architecture, increasing sleep fragmentation, decreasing superficial sleep (see Glossary) and, interestingly, increasing REM sleep (Chikadze et al., 2013).

Fish schooling structure and dynamics are also affected by hypoxic environments: individuals may swim further apart to maximise oxygen availability, or they may aggregate in smaller groups to reduce competition for limited oxygen (Domenici et al., 2013). Changes in group structure during the day may carry over and affect sleep, if for example, fish sleep in congregations to reduce the risk of predation but reach sleep sites in smaller groups in hypoxic areas. It would be interesting to explore the direct effect of the stressor (i.e. limited dissolved oxygen in the water) on fish sleep, as well as indirect effects resulting from factors such as group size.

### Carbon dioxide

Carbon dioxide has the potential to alter sleep behaviour. In mammals, blood pCO_2_ is a regulator of arousal, and it affects core clock gene expression, changing the intrinsic circadian clock on a molecular level (Adamovich et al., 2019). In fish, increased carbon dioxide levels in aquatic environments may lead to increased boldness and activity, and decreased behavioural lateralisation by interfering with neurotransmitters (Nilsson et al., 2012). This may have a direct effect on sleep behaviour, impacting clock genes and neurotransmitters regulating sleep/activity cycles as in mammals. Alternatively, it may have an indirect effect, whereby activity increases caused by increased carbon dioxide levels may increase the need for sleep and the energy conservation linked to sleep behaviour.

### Noise

Much of the research that investigates the effect of noise on sleep has been focused on humans and how traffic noise can affect human health (Pirrera et al., 2010). However, deleterious impacts of noise on sleeping behaviour have been documented in non-human animals as well (e.g. rats and birds; Grunst et al., 2022; Rabat, 2007; Slabbekoorn et al., 2018). The same is likely to be true in fish, where anthropogenic noise has been shown to cause increased stress (Mills et al., 2020). When exposed to seismic noise, Atlantic cod (*Gadus morhua*) reduce their activity and disrupt diurnal feeding cycles (Knaap et al., 2021). Relative to a control group not exposed to noise, European seabass (*Dicentrarchus labrax*) increase their swimming speed and depth in response to noise. They exhibit a greater behavioural difference during the night, relative to daytime behavioural changes (Neo et al., 2018). With increased noise at night from anthropogenic sources (e.g. boat noise, or noise generated from offshore windmill construction and use) (Andrew et al., 2002; Peng et al., 2015; Yoon et al., 2023), a better understanding of the consequences of noise-induced sleep disruption is vital.

### Food availability

Several research studies in non-fish species have found that the type and amount of food that an animal eats affects sleep, which generally increases after heavy feeding (Brown and Keene, 2023; Danguir and Nicolaidis, 1979; Keene et al., 2010; Lakhiani et al., 2023; MacFadyen et al., 1973; Murphy et al., 2016; Stahl et al., 1983). In fish, little is known about the direct role of diet and food availability on sleeping behaviour, but it is likely to play a role (Keene and Duboué, 2018). Indeed, fish need to continuously assess the costs and benefits of sleeping, which may come at the expense of losing an opportunity to forage; however, time spent asleep should decrease energy expenditure and thus foraging requirements. In cavefish, sleeping decreases when food is available and foraging increases (Jaggard et al., 2017). This is in contrast to surface fish and many other animals, which increase sleep with food availability. This may be linked to the specific adaptation of cavefish to extreme environments with a general low availability of food (Keene and Duboué, 2018).

### Risk of predation and parasitism

Predation risk may have particularly strong influences on sleep owing to the trade-offs between sleep and vigilance behaviour. In non-fish species, predator presence can either increase or decrease sleep behaviour: desert iguanas (*Dipsosaurus dorsalis*) sleep less when in close proximity to a predator (Revell and Hayes, 2009), but great tits (*Parus major*) increase sleep duration, potentially as a way to generally reduce activity and conserve energy (Stuber et al., 2014). Numerous factors, including the hunting strategy of the predator and the frequency and pattern of exposure, may influence responses to a predator. Additionally, factors that may increase or decrease the perceived risk associated with predators, including group size (Beauchamp, 2009; Lendrem, 1984; Maruyama et al., 2009) and available cover (Tisdale et al., 2018), may also influence the effect of predator exposure on sleep. Numerous questions about predator responses have been studied in active fish because there are many species that are tractable to study in the laboratory that have diverse predators; fish could also serve as a useful subject for understanding the influence of predators on sleep.

Although some work has been done on the effect of sleep on parasitism (see above; Bouchet, 1989; Johnson et al., 1995), the influence of parasites (or the threat of parasites) on sleep architecture is less well studied. In other non-fish species, sleep deprivation impairs the immune system, increasing the risk of parasitic infection (Ibarra-Coronado et al., 2015).

## Evolution of sleep

The apparent ubiquitousness of sleep across taxa makes the study of the evolution of sleep hugely compelling (Anafi et al., 2019; Aristakesyan, 2016). Sleep is responsive to both internal and external conditions, meaning that across phylogeny there is great variation in sleep architecture, timing and behaviour (Ungurean et al., 2020). However, there are many conserved aspects of sleep physiology and behaviour (as is evident from the behavioural criteria discussed above); this conservation is likely due to a shared or convergent evolutionary path (Keene and Duboue, 2018; Rattenborg and Ungurean, 2023). Early animals, such as the placozoans, having evolved locomotion would also have needed rest behaviour and a control system by which to govern this rest (Bringmann, 2018; Jaggard et al., 2021). With the evolution of the nervous system, animals gained sleep-inducing neurons, which are conserved across taxa, from cnidarians to mammals (Bringmann, 2018).

In the diversification of sleep and sleep states, more complex brains, specifically those of mammals, birds and potentially reptiles (Shein-Idelson et al., 2016), evolved the ability to undergo REM and non-REM sleep (Bringmann, 2018). REM sleep is characterised by particular brain and muscle activity, and the occurrence of REM only in certain taxa suggests its importance in some higher brain functions. REM and non-REM sleep can be distinguished using electroencephalograms to measure brain wave activity, but in species such as fish that do not exhibit typical REM sleep owing to physiological differences, using behavioural criteria has long been the sole method for the identification and distinction of sleep states versus resting states. However, researchers using florescence-based polysomnography to study brain activity in fish showed that young zebrafish also have two neurologically distinct sleep states (Blanco et al., 2023; Leung et al., 2019). These have been termed ‘propagating wave sleep’, consisting of a reduction in muscle tone and variability in heart rate, and ‘slow-bursting sleep’, which increases in duration in response to sleep deprivation. These states are analogous to mammalian REM and non-REM sleep, respectively. Similar active sleep states, showing parallels to REM sleep, are present in cephalopods (Medeiros et al., 2021) and *Drosophila* (Tainton-Heap et al., 2021), and so REM-like sleep may be more prevalent throughout taxa when studied using wider criteria that are not specific to mammalian and avian systems. The parallels between sleep signatures across taxa suggest that common ancestors sharing these neural signatures may have existed 450 million years ago (Leung et al., 2019).

Although understanding the similarities in sleeping across taxa is important, studying species in which individuals do not fulfil any of the behavioural criteria for sleep can be the most illuminating way to elucidate evolutionary relationships and constraints (Cirelli and Tononi, 2008; Siegel, 2008). Fish species such as mackerel, tuna and some sharks all exhibit constant activity – swimming and eating at all times of day (Bloch et al., 2013). They cannot cease swimming for more than a few moments, as they all share similar respiratory physiology: obligate ram gill ventilation (Bloch et al., 2013). They must continue swimming to respire and, as such, do not show any periods of quiescence. Despite this, two obligate-swimming shark species, *Mustelus lenticulatus* and *Squalus acanthias*, swim slower and cover less distance at night (Kelly et al., 2020b), indicating a period of lessened energy expenditure. This may represent the remnants of circadian rest rhythms or a behavioural response to darkness: if searching for prey becomes difficult, movement may be lessened to only that required to allow respiration. The leading hypothesis for how some animals can persist without sleeping ties in aspects of ecology and neuroscience (Aulsebrook et al., 2016; Kavanau, 2004): when sensory processing demands are reduced, there is less need to sleep, and the memory processing aspect of sleep is inconsequential as a result of monotonous movement and routine (Kavanau, 1998, 2004). This is consistent with the fact that many apparently sleepless fish species are either pelagic or more active at night, thus experiencing less visual sensory input throughout the day than a diurnal fish in a rapidly changing environment (Kavanau, 2001). However, it is also possible that fish species that do not fulfil the behavioural criteria for sleep could undergo another form of rest, allowing movement alongside decreased sensory responsiveness, similar to the unihemispheric sleep (see Glossary) exhibited in some marine mammals and birds (Konadhode et al., 2016). Seemingly sleepless species could also use other sleep tactics, such as localised sleep in the brain, where the animal is behaviourally awake while some neurons go ‘offline’ (Vyazovskiy et al., 2011). Similarly, microsleeping, where the brain is ‘asleep’ for seconds at a time, allows maintenance of a behaviourally awake phenotype, while obtaining sleep (Libourel et al., 2023). The possibility of localised sleep opens new avenues of research in obligate swimming species, which could achieve sleep while remaining active.

## Conclusions and future directions

Although numerous studies describe phylogenetically diverse fish species engaging in behaviours associated with sleep, far fewer studies definitively demonstrate the fulfilment of all criteria of sleep. Aspects of sleep-like behaviour – particularly circadian rhythm – are well understood; however, it is important to bridge the gap between describing resting behaviours and true sleep, to fully understand the importance of sleep in fish and to begin to take the research further towards understanding the ecological implications of sleep. Fish sleep research has only gained traction in the past few decades, as an increase in studies on model species for research on sleep functions and disorders has broadened perspectives on resting states. Although there are a multitude of intriguing and exciting directions to explore, we highlight four priority areas for additional study.

First, validation of the occurrence of sleep in additional species. At present, most of our knowledge on fish sleep comes from a small subset of the more than 30,000 species of fish. Study of sleep in a broader range of fish taxa across diverse habitats will increase our general knowledge of sleep in fishes, increase our ability to derive testable hypotheses, and allow us to identify unique study systems.

Second, the effects of environmental factors on sleep architecture. Fish are continuously exposed to shifting biotic and abiotic conditions, yet we know very little about how this affects sleep, one of the most fundamental and seemingly ubiquitous dimensions of their biology.

Third, the effects of sleep disruption on behaviour and physiology at the whole-organism level. Research into the effects of the environment on fish physiology, behaviour and ecology have focused exclusively on the waking period, overlooking the effects of sleep, which are known to drastically alter physiology and behaviour in mammals and birds.

And fourth, the effects of sleep disruption on sensitivity to environmental factors. Any effects of sleep disruption may have important yet unnoticed implications for how fish respond to ongoing environmental change. For example, sleep may alter aspects of metabolism or the primary stress response, which are believed to directly affect sensitivity to factors such as temperature and hypoxia. Similarly, if sleep is involved in tolerance to stressors during the waking period, changing environments may evoke plastic and adaptive changes in sleep architecture. Alternatively, the effects of sleep disruption on the expression of behavioural or physiological traits may alter trait variation or repeatability, influencing the extent to which they can adapt or be targeted by selection.

There is also an absence of investigations that cover both laboratory and field studies. Combining these would open a new avenue for moving research beyond rest behaviour to understanding sleep. We could then move on to investigating the ecological relevance of sleep: how does an organism's environment affect how it sleeps, and how does an organism's sleep affect how it interacts with its environment? Currently, the potential for various natural and anthropogenic factors to evoke a plastic response in the sleep behaviour of fish is completely unknown, and investigating this would both add additional knowledge regarding sleep behaviour in a three-dimensional environment, and give a baseline for understanding the potential for natural and anthropogenic influences to alter sleep. Determining the potential implications of such changes will be of great interest going forwards.

## Supplementary Material

10.1242/jexbio.247138_sup1 Supplementary information

Table S1. Table listing all relevant papers filtered through the literature search and subsequent screening process, including the search number (details listed in Table 1), Authors, Title, DOI, Year of publication, and Type of paper (primary research or Review).
