# Supplementary material for: The interplay between sleep and ecophysiology, behaviour and responses to environmental change in fish
Source: J Exp Biol. 2024 Jun 11;227(11):jeb247138. doi: 10.1242/jeb.247138 (PMC11213526; doi:10.1242/jeb.247138)
Supplement: Supplementary information [file jexbio-227-247138-s1.pdf]

Table S1. Table listing all relevant papers filtered through the literature search and subsequent screening process, including the search number (details listed in Table 1), Authors, Title, DOI, Year of publication, and Type of paper (primary research or Review).

Available for download at
https://journals.biologists.com/jeb/article-lookup/doi/10.1242/jeb.247138#supplementary-data
